# Exercise enhances mitochondrial fission and mitophagy to improve myopathy following critical limb ischemia in elderly mice via the PGC1a/FNDC5/irisin pathway

**DOI:** 10.1186/s13395-020-00245-2

**Published:** 2020-09-15

**Authors:** Wuyang He, Peng Wang, Qingwei Chen, Chunqiu Li

**Affiliations:** 1 Department of Oncology, The Second Affiliated Hospital of Chongqing Medical University, Chongqing, China; 2 Department of Geriatric Cardiology, The Second Affiliated Hospital of Chongqing Medical University, No. 76 Linjiang Road, Chongqing, 400010 China; 3 Department of Geriatric Cardiology, The Central Hospital of Fuling District, Chongqing, China

**Keywords:** Exercise, Critical limb ischemia, Skeletal muscle, Mitochondrial turnover, PGC1a

## Abstract

**Background:**

Elderly populations are susceptible to critical limb ischemia (CLI), but conventional treatments cannot significantly decrease amputation and mortality. Although exercise is an effective “non-pharmacological medicine” targeting mitochondria to improve skeletal muscle function, few studies have focused on the application of exercise in CLI.

**Methods:**

Elderly male C57BL/6 mice (14 months old) were used to establish a CLI model to assess the effect of exercise on perfusion, performance recovery, apoptosis, mitochondrial function, and mitochondrial turnover in gastrocnemius muscle. The potential underlying mechanism mediated by PGC1a/FNDC5/irisin was confirmed in hypoxic and nutrient-deprived myotubes undergoing electrical pulse stimuli (EPS).

**Results:**

Exercise significantly accelerated the perfusion recovery and exercise performance in ischemic limbs following CLI. Exercise improved the mitochondrial membrane potential and total ATP production and decreased apoptosis in the ischemic limbs. Exercise increased the formation of mitochondrial derived vesicle-like structures and decreased the mitochondrial length in the ischemic limbs, accompanied by upregulated PGC1a/FNDC5/irisin expression. In vitro, PGC1a/FNDC5/irisin downregulation decreased EPS-elevated PINK1, Parkin, DRP1, and LC3B mRNA levels. The irisin levels in the culture medium were correlated with the expression of mitochondrial fission and mitophagy markers in myotubes.

**Conclusion:**

Exercise enhanced mitochondrial fission and selective autophagy to promote the recovery of myopathy after CLI in elderly mice through the PGC1a/FNDC5/irisin pathway, supporting the efficacy of exercise therapy in elderly individuals with CLI and demonstrating the potential of targeting PGC1a/FNDC5/irisin as a new strategy for the treatment of CLI.

## Highlights

- Exercise accelerated the improvement of skeletal muscle function and the perfusion recovery in a CLI model of elderly mice.
- Exercise increased the formation of MDV-like structures and decreased mitochondrial length in ischemic limbs accompanied by elevated PGC1a/FNDC5/irisin expression.
- Downregulated PGC1a/FNDC5/irisin expression reduced the EPS-increased mitochondrial turnover and ATP production in ischemic myotubes.
- Inhibition of autophagy caused the upregulation of PGC1a/FNDC5/irisin accompanied by PINK1/PARKIN.
- Irisin levels in the culture medium were significantly related to mitochondrial turnover markers.

## Background

The prevalence of peripheral artery disease (PAD) increases substantially with age, and thus, elderly individuals are more susceptible to critical limb ischemia (CLI), a severe manifestation of PAD, which results in poor prognosis [1, 29]. Despite the application of surgical and endovascular revascularization in PAD, ischemic myopathy is often ignored, and the risk of amputation and mortality in CLI patients cannot be effectively decreased [25]. For elderly patients who cannot be given conventional interventions due to unfavorable anatomic characteristics of the lesions, comorbidity, and frailty status [6, 25], the treatment of myopathy is detrimental for limb salvage and survival in elderly patients with CLI [13]. Mitochondria are organelles with a major role in enhancing skeletal function since they not only produce ATP to meet energy demands but also regulate cell apoptosis and calcium retention of skeletal muscle [7, 26].

The maintenance of metabolic homeostasis depends on the dynamic process of mitochondrial biogenesis, mitochondrial dynamics (fusion and fission), and mitophagy. Once homeostasis is disturbed, the balance may shift to fission and mitophagy to achieve the selective clearance of damaged organelles [11]. Given the decreased expression of mitochondrial protein and mRNA observed in aged skeletal muscle [18], mitochondrial adaptation to severe stress, such as CLI, can be decreased by aging.

Exercise training is an economic and efficient non-pharmacological intervention targeting mitochondria that induces beneficial mitochondrial adaptations, increasing mitochondrial quality and content [22]. Exercise has been widely applied in the treatment of claudication, which results in the improvement of exercise performance and quality of life [10]. Exercise therapy even results in a more robust improvement in peak walking time than stent placement [24]. More extensive mitochondriopathy has been observed in the skeletal muscle of CLI patients than claudicants [28]. However, the application and mechanism of exercise therapy for the treatment of CLI remains to be investigated.

Exercise-induced mitochondrial adaptations in skeletal muscle converge on peroxisome proliferator-activated receptor gamma coactivator-1 alpha (PGC1a), which activates the downstream factor FNDC5 in the membrane of skeletal muscle cells, synthesizing and encoding irisin [27]. FNDC5/irisin is a promising intervention target in the treatment of metabolic disease based on its role in browning white adipocyte tissue [2]. The close relationship of FNDC5/irisin with mitochondrial genes and proteins that regulate mitochondrial function has recently been reported [5, 32]. Here we hypothesize that exercise could improve mitochondrial health to alleviate CLI-induced myopathy in elderly populations via the PGC1a/FNDC5/irisin signaling pathway. To test this hypothesis, we established a CLI model in elderly mice to measure the efficacy of exercise for the treatment of CLI in elderly animals, and we investigated the underlying mechanism in vitro.

## Materials and methods

### Animals

Sixty elderly male C57BL/6 mice (14 months old, weighting 29–33 g) were purchased from the animal laboratory of Chongqing Medical University. Five mice were kept in a cage with free access to water and food, a controlled room temperature of 22 ± 2 °C, and a 12-h light/dark cycle. All procedures were approved by the Animal Care and Use Committee of Chongqing Medical University and were conducted according to the ARRIVE guidelines.

### Bilateral CLI model

After acclimatizing for 1 month, all mice underwent isoflurane anesthesia (2%) using a mask. A single researcher ligated and excised the left femoral artery from its origin below the inguinal ligament. The inferior epigastric, lateral circumflex, and superficial epigastric artery branches of the femoral artery were also dissected and excised. The right limb underwent the same incision except for ligation and excision of the femoral arteries, and its collateral vessels could act as a sham-operated control. During the surgery, 7 mice died because of massive hemorrhage, anesthesia, or glossoptosis.

All mice were randomly separated into exercised mice (*n* = 27) and sedentary mice (*n* = 26). Exercised mice underwent exercise intervention and were sacrificed on the 30th day after surgery; their left limbs were selected for the CLI + exercise group, and the right limbs were selected for the control + exercise group. Sedentary mice could freely move within the cage and were sacrificed 30 days after the operation; the left limbs were selected for the CLI + sedentary group, and the right limbs were chosen as the control + sedentary group.

### Assessment of hindlimb blood flow

The blood flow of the ischemic limbs and the controls was evaluated by moorLDI2-HIR (Moor Instruments, UK) under anesthesia on day 7, day 14, day 21, and day 28 after artery ligation and excision. The ratio of ischemic (left)/control (right) limb blood flow of each mouse was calculated.

### Exercise protocol

After recovery from the wound for 1 week, the exercised mice underwent moderate exercise intervention through an animal treadmill (Zhongshi, Inc.) for 5 days/week. The speed of treadmill running was set at 10 m/min (75% VO2 max) as previously described [8]. Warm-up exercise (4 m/min for 2 min) was applied prior to each exercise protocol. The adaptation training was conducted during the 2nd week, and the mice ran in the treadmill with a belt speed set at 6 m/min for 15 min on day 1 and day 2, for 20 min on day 3 and day 4, and for 30 min on day 5. The mice underwent a moderate exercise protocol during the 3rd and 4th weeks and ran in the treadmill at a speed of 10 m/min for 1 h. When the mice were exhausted (could not reach the belt speed), they were allowed to rest for 30–60 min and then continued the protocol. The length of time to exhaustion was recorded per week. All mice were sacrificed on day 30 (4 days after the final exercise training). The gastrocnemius muscles were dissected and immediately analyzed for mitochondrial function, and the rest of the tissue was stored in liquid nitrogen for further assessment.

### Determination of the tissue damage and function scores

All mice were weighed every week using electronic scales. The necrosis score and the function score of the lower limbs were determined by two different single-blinded investigators using validated methods on day 0, day 7, day 14, day 21, and day 28 after the operation [8, 20]. For the necrosis score, the ischemic limb with no necrosis was defined as grade 0; necrosis limited to toes was defined as grade 1; necrosis extending to the dorsum pedis was defined as grade 2; necrosis extending to the crus was defined as grade 3; and necrosis extending to the thigh or complete limb necrosis was defined as grade 4; a toe has been spontaneously amputated. For the function score, the limb with the normal function was defined as 0, the presence of plantar flexion but no toe flexion was defined as 1, the inability to achieve toe flexion was defined as 2, and dragging limbs were defined as 3.

### Histological analysis: hematoxylin and eosin, CD31, and TUNEL

Gastrocnemius muscle tissues were embedded in paraffin after being fixed in paraformaldehyde fixative, which was sliced into 5-μm-thick samples. Following xylene dewaxing and dehydration in ethanol, hematoxylin and eosin staining was applied to the specimens on glass slides, which were evaluated via bright field microscopy (Thermo Scientific).

These paraffin-embedded skeletal muscle tissues were immunohistochemically stained using a CD31-specific antibody (Abcam, Cambridge, MA). Image-Pro Plus 6.0 was used to analyze the number of CD31-positive cells in each sample. During the procedure, five fields from each group were randomly chosen, and the average number of CD31-positive cells was recorded.

The apoptosis of skeletal muscle cells was measured by immunohistochemistry using a Colorimetric TUNEL Apoptosis Assay Kit (Beyotime). The mean value of TUNEL-positive cells was determined through the analysis of 5 randomly chosen regions under a light microscope.

### Mitochondrial function

#### Preparation of mitochondria from skeletal muscle

Fresh gastrocnemius muscle tissues were homogenized by a glass homogenizer on ice for 1 h after sacrifice according to the guidelines of the tissue mitochondria isolation kit (Beyotime, China, c3606). After the homogenate was centrifuged at 600 *g*/min for 10 min, the divided supernatant was further centrifuged at 11000 *g*/min for 15 min. The mitochondrial pellet was resuspended in mitochondrial isolation buffer (Beyotime, Shanghai, China).

#### Mitochondrial membrane potential (∆Ψm)

The mitochondrial pellet was stained with JC-1 according to the guidelines of the mitochondrial membrane potential (∆Ψm) assay kit with JC-1(Beyotime, China, c2006). The absorption of monomer (fluorescence excitation was set at 490 nm; fluorescence excitation was set at 530 nm) and J-aggregates (fluorescence excitation was set at 525 nm; fluorescence excitation was set at 590 nm) was measured by a modular multitechnology microplate reader (Thermo Scientific™ Varioskan™ LUX) using fluorescence spectrophotometry according to the manufacturer’s protocol. The ratio of J-aggregates/monomer reflects the variation of mitochondrial membrane potential among groups.

#### ATP assessment

After the fresh skeletal muscle tissue was homogenized by a homogenizer on ice, the supernatant was collected, and adenosine 5′-triphosphate (ATP) was measured according to the guidelines of the ATP Assay Kit (Beyotime, China, S0026) through a modular multitechnology microplate reader (Thermo Scientific™ Varioskan™ LUX) using chemiluminescence. The ATP protein concentration was measured by an Enhanced BCA Protein Assay kit (Beyotime, China, P0010S).

### Transmission electron microscopy

The fresh gastrocnemius muscle was cut into five tissue blocks (1 mm^3^). After fixation, dehydration, infiltration, and dying with uranyl acetate and lead citrate as previously described [36], the samples were observed with transmission electron microscopy (Hitachi-7700, Japan) by two independent investigators who were single blinded to the experiment. Ten random fields of images were observed in each section. The formation of mitochondrial derived vesicles was identified as previously described [3, 19].

### Cell culture and transfection

C2C12 myoblasts at passages 4 ~ 10 (Zhongqiao Biotech, Shanghai) were maintained in Dulbecco’s modified Eagle’s medium (DMEM, Gibco, NY, USA) supplemented with 10% FBS and 1% antibiotics (100 μg/ml streptomycin and 100 U/ml penicillin) in a 37 °C incubator with 5% CO_2_. The differentiation of myoblasts was induced in differentiation medium (DMEM supplemented with 5% horse serum (Biological Industry) and 1% antibiotics) once 80% ~ 90% confluence was reached. The differentiation medium was changed every day until the C2C12 myotubes were induced on the 6th ~ 7th day of differentiation.

On the last day of differentiation, C2C12 myotubes were incubated with siRNA against PGC1a (Ppargc1a-mus-306: GUAGCGACCAAUCGGAAAUTT; AUUUCCGAUUGGUCGCUACTT) for 6 h according to the standard protocol (GenePharma, Inc.). A sequence (5′-UUCUCCGAACGUGUCACGUTT-3′; 5′-ACGUGACACGUUCGGAGAATT-3′) was chosen as the negative control. After incubation with siRNA for 6 h, the culture medium was immediately changed to hypoxia and nutrient deprivation medium.

The incubation of C2C12 myotube with 2.5 mM 3-methyladenine (3MA) (3MA, Selleck, Inc) for 24 h was conducted according to the manufacturer’s protocol.

### The establishment of the hypoxia and nutrient deprivation model

A previously described, the hypoxia and nutrient deprivation model was used to mimic the ischemic environment in vivo [20]. Given that cobalt chloride (CoCl2) has been widely used to establish sustainable chemical hypoxic preconditioning [23], myotubes incubated with serum-free DMEM supplemented with 250 μM CoCl2 for 24 h were used to generate ischemic myotubes in vitro based on the 50% decrease of cell viability in CCK8 assays (data not shown).

### Electrical pulse stimulation (EPS)

The ischemic C2C12 myotubes cultured in 6-well dishes were placed into a C-Dish (IonOptix, Milton, MA), in which electrical stimulation (1 Hz, 2 ms, 11.5 V) was conducted by C-Pace (C-Pace 100; IonOptix) for 24 h according to the manufacturer’s protocol [15]. The culture medium was switched to serum-free DMEM to eliminate the effect of serum on the assessment of irisin levels.

### Real-time PCR

The PCR primers for PGC1a/FNDC5, mitophagy markers (LC3B), and markers of mitochondrial fission (including DRP1, FIS1) were listed in Table 1. RNA was extracted from the skeletal muscle and the C2C12 myotubes using TRIzol Reagent (TaKaRa, Inc.). RNA denaturation and cDNA reverse transcription were performed with a PrimeScript RT Reagent Kit (TaKaRa, Inc.). The target genes were assessed using quantitative real-time PCR, and three duplicates were set for each sample. The 2 − △△CT method was performed to analyze the data.

**Table 1:** The list of PCR primers

Gene name	Sequence 5′–3′ (forward)	Sequence 5′–3′ (reverse)
PGC1a	tat gga gtg aca tag agt gtg ct	ccacttcaatcc acc cag aaa g
FNDC5	ttgccatctctcagc aga aga	ggcctgcacatggac gat a
DRP1	act gat tcaatccgt gat gag t	gta acc tat tcagggtcc tag c
FIS1	gacatccgc aga ggcatc gtg	cag ctccttggcctggttgtt c
LC3B	cgtcctggacaagaccaagtt c	gcaagcgccgtctgattatc
PINK1	gca gca gtc agc agc cac tc	agc ctc aca ctc cag gtt agc c
PARKIN	aag gaa gtg gtt gct aag cga cag	atg act tct cct ccg tgg tct ctg
GAPDH	ggttgtctcctgcgactt ca	tggtccagggtttct tac tcc

### Western blot analysis

Western blotting was used to assess the protein expression of each marker. Briefly, the harvested skeletal muscle was homogenized in RIPA buffer (Beyotime, Inc.) supplemented with the protein inhibitor PMSF. Equal amounts of protein from each sample were subjected to 10% SDS-PAGE and then transferred to PVDF membranes (Roche). After the membranes were blocked with 5% nonfat dry milk for 2 h at room temperature, they were incubated with primary antibodies against PGC1a (1:500, Zenbio), FNDC5 (1:500, Proteintech), MFN2 (1:500, Zenbio), PINK1 (1:500, Zenbio), PARKIN (1:500, Zenbio), DRP1 (1:500, Zenbio), FIS (1:500, Proteintech), and LC3B (1:500, Proteintech) at 4 °C overnight. Then, the membranes were washed 4 times using PBST and incubated with the anti-mouse or anti-rabbit secondary antibody (1:5000, Zhongshan, Inc.) for 1 h at room temperature. After these membranes were washed in PBST 4 times, bands were detected with an enhanced chemiluminescence kit (EpiZyme™). ImageJ software was used to quantify the protein bands.

### Elisa

The irisin concentrations of murine serum collected before sacrifice and culture medium after EPS were measured via standard ELISAs (MB-5653B, Mbbiology, China) according to the manufacturer’s guidelines.

### Statistical analysis

The normality of continuous variables was tested by the Kolmogorov-Smirnov test. ANOVA was used to compare the normally distributed variables among groups. The comparison of skewed variables was conducted by the Mann-Whitney *U* test. The data are shown as the mean ± standard deviation (SD) or medians with interquartile ranges for quantitative values. The linear relationship between irisin levels and the expression of mitochondrial turnover markers was measured by Spearman correlation analysis. All the data were analyzed by IBM SPSS Statistics version 25.0 and GraphPad Prism 6, and *p* values < 0.05 were considered significant.

## Results

### The effects of exercise on blood flow and functional recovery after CLI

**Fig. 1:**
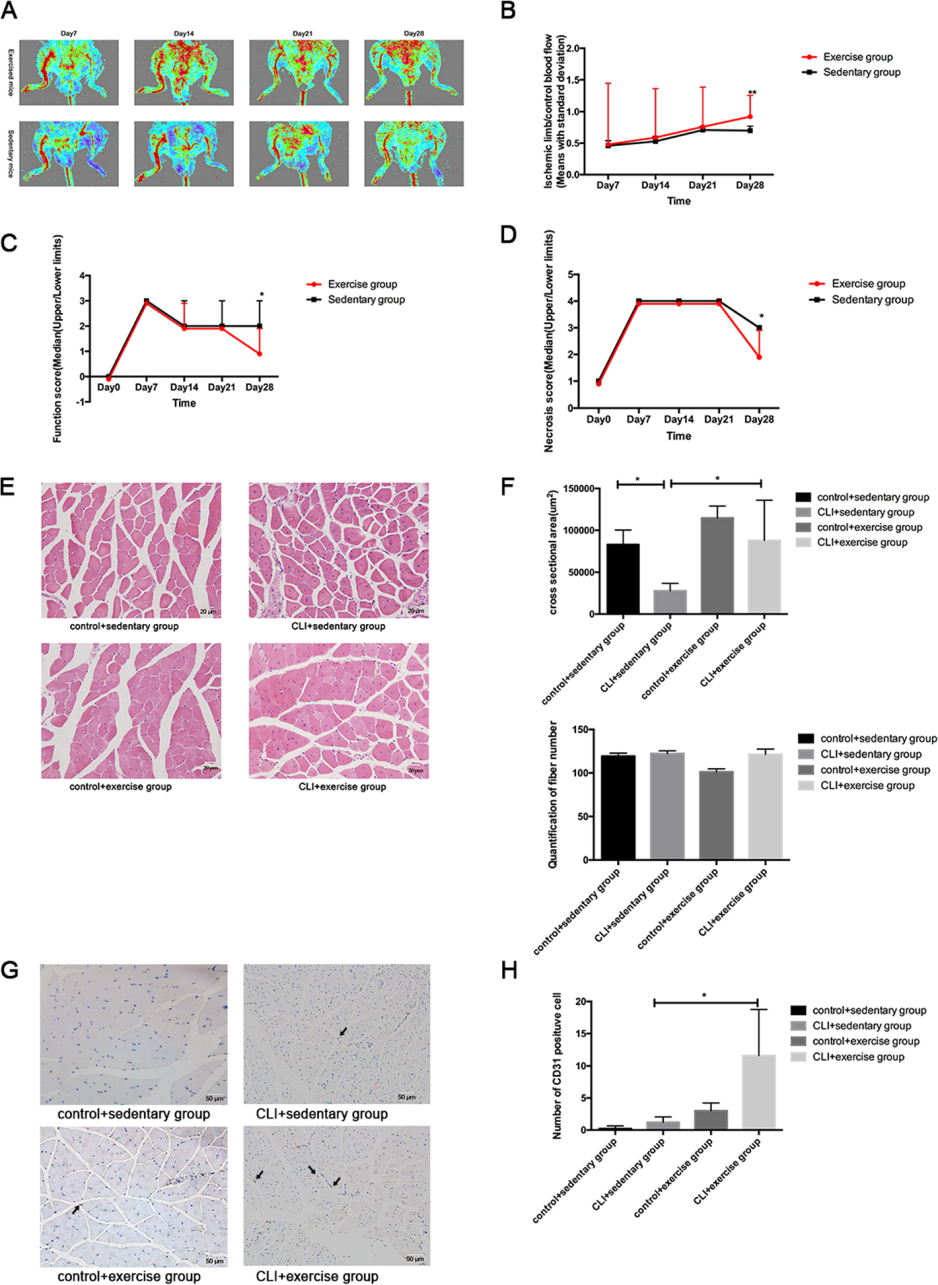
The effects of exercise on the blood flow and functional recovery following CLI. **a** The perfusion of lower limbs in exercised and sedentary mice. **b** The ischemic/control blood flow of exercised mice (red line, *n* = 27) and sedentary mice (black line, *n* = 26) on different days. The results were displayed as means with standard derivation. The comparison of function (**c**) and necrosis scores (**d**) between exercised mice (red line, *n* = 27) and sedentary mice (black line, *n* = 26) was conducted. The results were shown as median (upper/lower limits). **e** The hematoxylin-eosin staining of four groups (*n* = 3 samples/group). Magnification × 400. **f** The comparison of cross-sectional area was used by ANOVA (*n* = 3 samples/group) and the comparison of quantification of fiber numbers was conducted by the Mann-Whitney *U* test (*n* = 3 samples/group). **g** The number of CD31-positive cells (indicated by the black arrowheads) was compared among four groups (*n* = 3 samples/group). Magnification × 200. **h** The Mann-Whitney *U* test was used to compare the difference in the number of CD31-positive cells among groups. *p < 0.05, **p < 0.01

None of the mice died during the 4-week exercise training. As shown in Fig. 1, the limb perfusion of all the mice began to recover from day 14 after the surgery. On day 28, the ischemic limb/control blood flow of the exercised mice was significantly higher than that of the sedentary mice (*p* < 0.01), indicating that exercise could accelerate the perfusion recovery (Fig. 1a, b). The mean values of both the function and damage scores were significantly lower in the exercise group than the sedentary group on day 28 (both *p* < 0.5), suggesting that exercise can enhance the recovery of limb function and tissue damage (Fig. 1c, d).

According to the hematoxylin and eosin staining, the sedentary ischemic limbs presented disarranged fibers, smaller cross-sectional areas, more rounded shapes with central nuclei, wider intermuscular spaces, expanded and rupture of capillaries, and more inflammatory cell infiltration than the control limbs (Fig. 1e, f). Exercise could decrease the ischemic features of skeletal muscle based on the decreased intermuscular spaces, more arranged fibers, bigger cross-sectional area, and the formation of myoblasts and new capillaries in the CLI + exercise group than the CLI + sedentary group (Fig. 1e, f). The fiber numbers of the ischemic limbs were also elevated by exercise, although the difference has not reached the significance (Fig. 1f).

As CD31 is a marker of endothelial angiogenesis, the difference in the numbers of CD31-positive cells was not significant between the sedentary ischemic limbs and controls (*p* > 0.05). Exercise promoted endothelial angiogenesis, as indicated by the increased numbers of CD31-positive cells in the CLI + exercise group compared with the CLI + sedentary group (*p* < 0.05) (Fig. 1g, h).

### The effects of exercise on the apoptosis and mitochondrial function of skeletal muscle

**Fig. 2:**
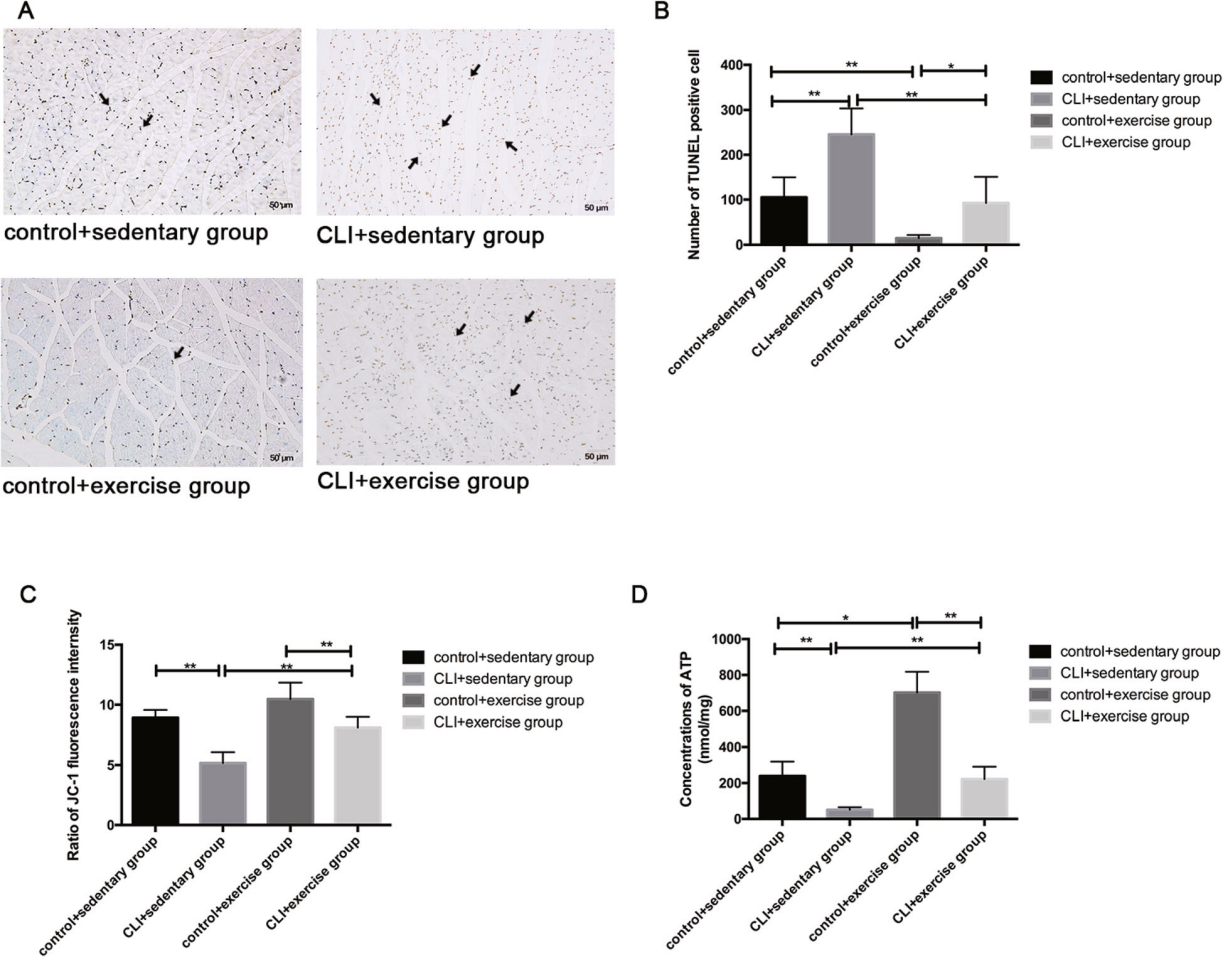
The effects of exercise on the apoptosis and mitochondrial function of skeletal muscle. **a** The number of TUNEL-positive cells (indicated by the black arrowheads) was counted in four groups (*n* = 3 samples/group). Magnification × 200. **b** The results were expressed as means with standard derivation. The comparison of mitochondrial membrane potential (∆Ψm) (**c**) and ATP concentrations (**d**) among groups was conducted by the Mann-Whitney *U* test (*n* = 15 samples/group). *p < 0.05, **p < 0.01

The apoptosis of skeletal muscle was assessed by TUNEL assays. The number of TUNEL-positive cells increased in the sedentary ischemic limbs compared with the controls (*p* < 0.01) (Fig. 2a, b). The CLI + exercise group had decreased TUNEL-positive cells compared with the CLI + sedentary group (*p* < 0.01) (Fig. 2a, b), suggesting that exercise could inhibit the apoptosis of skeletal muscle.

The mitochondrial membrane potential and total ATP content significantly decreased in the ischemic limbs following CLI compared with the control limbs (both *p* < 0.01) (Fig. 2c, d). Exercise training increased the mitochondrial membrane potential and the total ATP production in the ischemic limbs based on the comparison between the exercised ischemic limbs and the sedentary ischemic limbs (both *p* < 0.01) (Fig. 2c, d).

### The effects of exercise on mitochondria and myofibrils

**Fig. 3:**
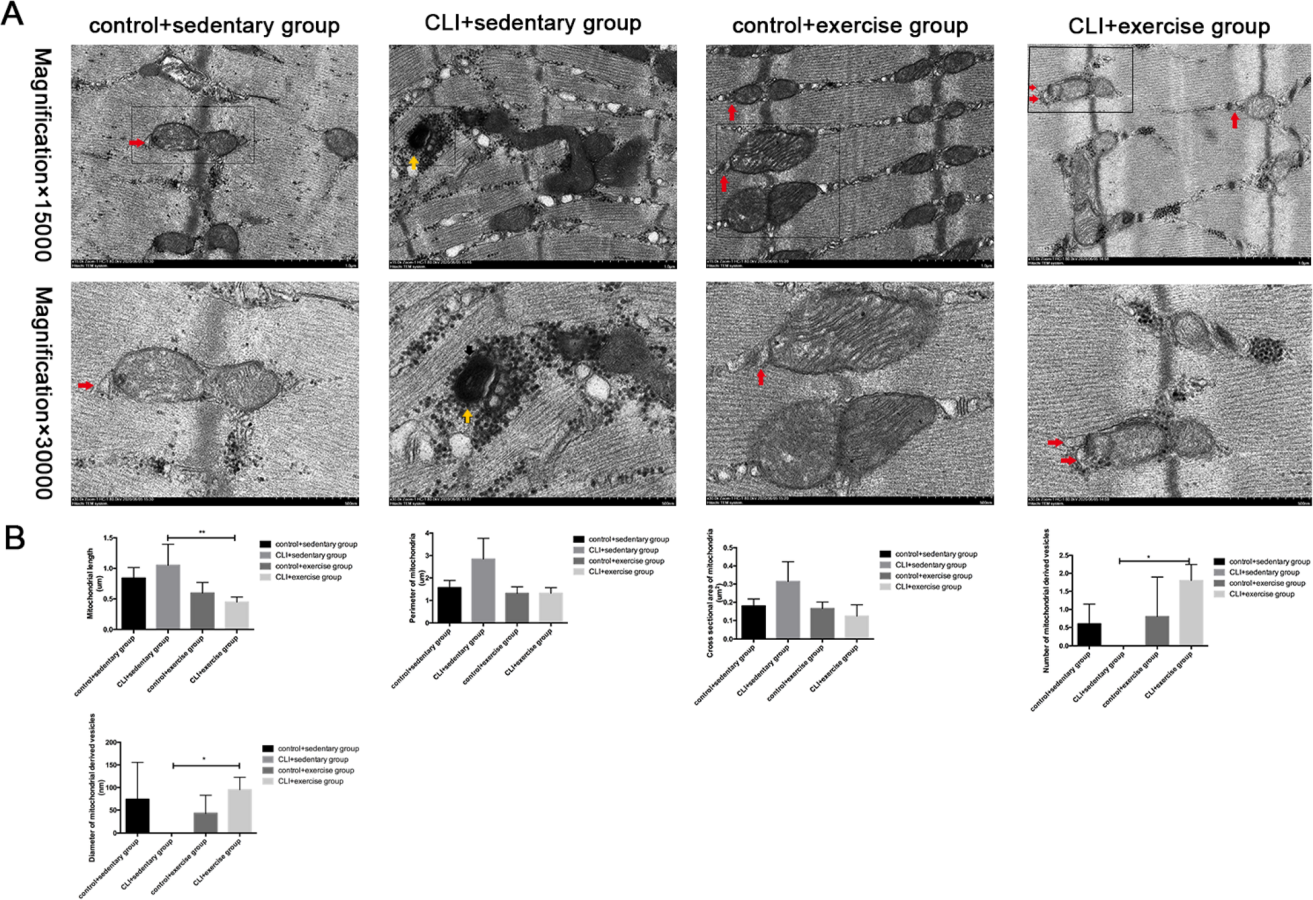
The effect of exercise on mitochondria and myofibrils. **a** The formation of mitochondrial derived vesicle-like structures (indicated by the red arrow) was shown in the CLI + exercise group and the myeloid body (indicated by the yellow arrow) was found in the CLI + sedentary group. The mitochondrial length, the cross-sectional area and perimeter of mitochondria, number of mitochondrial derived vesicles, and the diameter of mitochondrial derived vesicles were quantified using image-pro plus 6.0 (*n* = 3 samples/group). Magnification × 15,000. **b** The comparison of mitochondrial length among 4 groups was conducted using ANOVA. The cross-sectional area and perimeter of mitochondria, number of mitochondrial derived vesicles, and diameter of mitochondrial derived vesicles were compared via the Mann-Whitney *U* test, *p < 0.05, **p<0.01

Transmission electron microscopy demonstrated that the sedentary ischemic limbs had Z line derangement, discontinuous H zones, and more fractures of the muscle sarcomeres than the control limbs (Fig. 3a). The abnormal elongated intermyofibrillar mitochondria, the myeloid body formed by dysfunctional mitochondria, and more expanded sarcoplasmic reticulum were assessed in the CLI + sedentary group. However, more regular Z lines and continuous H zones were also shown in the CLI + exercise group than the CLI + sedentary group (Fig. 3a).

In addition, the intermyofibrillar mitochondria in the CLI + exercise group had shorter mitochondrial length, smaller cross-sectional area, and perimeter (Fig. 3a, b). The increased formation of mitochondrial derived vesicle (MDV)-like structures, which are contiguous to mitochondrial membrane, can be observed in ischemic limbs after exercise training (Fig. 3). There were no significant differences in the number of MDVs between the control + exercise group and the control + sedentary group.

### The effects of exercise on the markers of mitochondrial dynamics and mitophagy

**Fig. 4:**
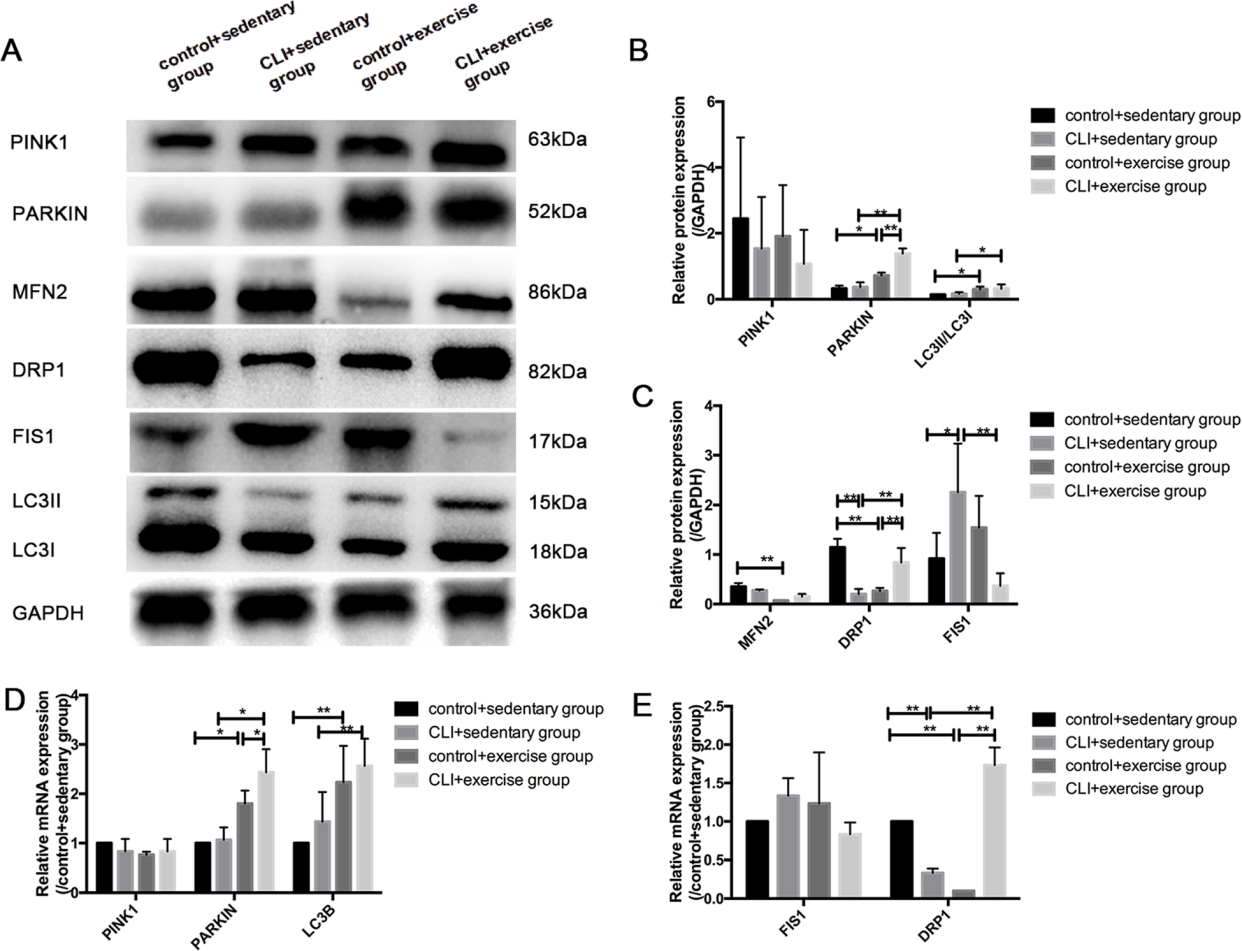
The effect of exercise on mitochondrial turnover. **a** Western blotting of PINK1, PARKIN, MFN2, DRP1, FIS1, LC3B, and GAPDH was chosen as the loading control. **b** Quantification of relative protein levels of mitophagy markers. The expression of LC3B was shown as LC3II/LC3I protein levels. **c** Quantification of protein levels of mitochondrial dynamic markers. Results were shown as means with standard derivation (SD). **d** The relative mRNA expression of mitophagy markers. **e** The relative mRNA expression of mitochondrial turnover markers. *n* = 5 samples/group, *p < 0.05, **p < 0.01

In the context of mitochondrial fission markers (Fig. 4a–e), the protein and mRNA expression of DRP1 were significantly decreased in the sedentary ischemic limbs compared with the controls (both *p* < 0.01). The expression of DRP1 at the protein and mRNA levels decreased in the control + exercise group compared with the control + sedentary group but increased in the CLI + exercise group compared with the CLI + sedentary group (all *p* < 0.01). In contrast to the change in DRP1 among the groups, FIS1 protein expression was elevated in the sedentary ischemic limbs compared with the controls (*p* < 0.05). Exercise significantly decreased the expression of FIS1 in the exercised ischemic limbs compared with the sedentary ischemic limbs (*p* < 0.01), while the effect of exercise on the control limbs was not significant. For the mitochondrial fusion marker, the protein expression of MFN2 decreased in the control + exercise group compared with the control + sedentary group (*p* < 0.05), but the impact of exercise on the ischemic limbs was not significant.

Regarding the markers of mitophagy (Fig. 4a–d), the mRNA and protein expression of PARKIN and LC3B increased in both the ischemic and control limbs in response to exercise training (all *p* < 0.05), although the difference between the ischemic limbs and the controls was not significant. As the exercise-induced elevation in PARKIN expression grows faster in ischemic limbs than that of control limbs, the CLI + exercise group had significantly higher PARKIN expression compared with the control + exercise group.

### The effects of exercise on the expression of PGC1a/FNDC5/irisin

**Fig. 5:**
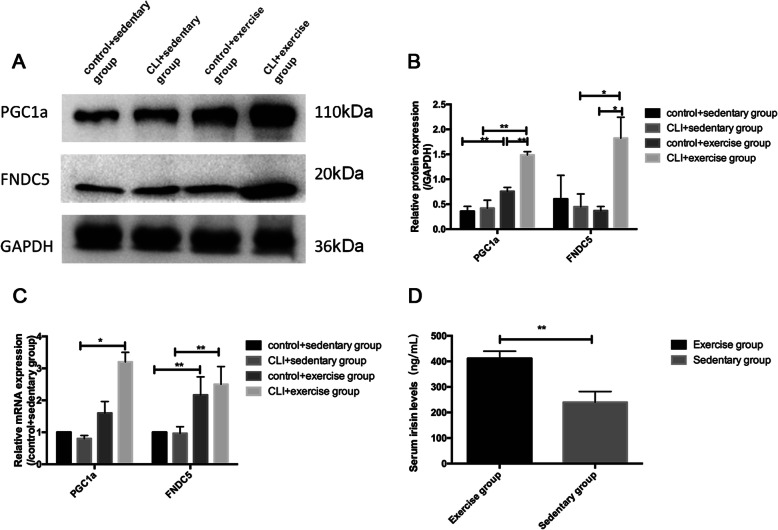
The effect of exercise on the expression of PGC1a/FNDC5/irisin. **a** Western blotting of PGC1a, FNDC5, and GAPDH was set as the loading control. *n* = 5 samples/group. **b** Quantification of PGC1a and FNDC5 protein levels. **c** Relative mRNA expressions of PGC1a and FNDC5 among groups. *n* = 5 samples/group. **d** Serum irisin levels of exercise and sedentary mice. *n* = 15 mice/group. *p < 0.05, **p < 0.01

While the difference in the protein and mRNA expression of PGC1a and FNDC5 was not significant between the sedentary ischemic limbs and the controls, exercise significantly elevated the expression of PGC1a/FNDC5 in both the ischemic and control limbs (both *p* < 0.05) (Fig. 5a–c). As the exercise-increased protein expression of PGC1a elevates faster in ischemic limbs than that of control limbs, the PGC1a expression was significantly increased in the CLI + exercise group compared with the control + exercise group (Fig. 5b, c). In parallel with the exercise-induced expression of PGC1a/FNDC5, the serum irisin levels were significantly elevated in the exercised CLI mice compared with the sedentary mice (*p* < 0.05) (Fig. 5d).

### The relationship of PGC1a/FNDC5/irisin with mitochondrial fission and mitophagy

**Fig. 6:**
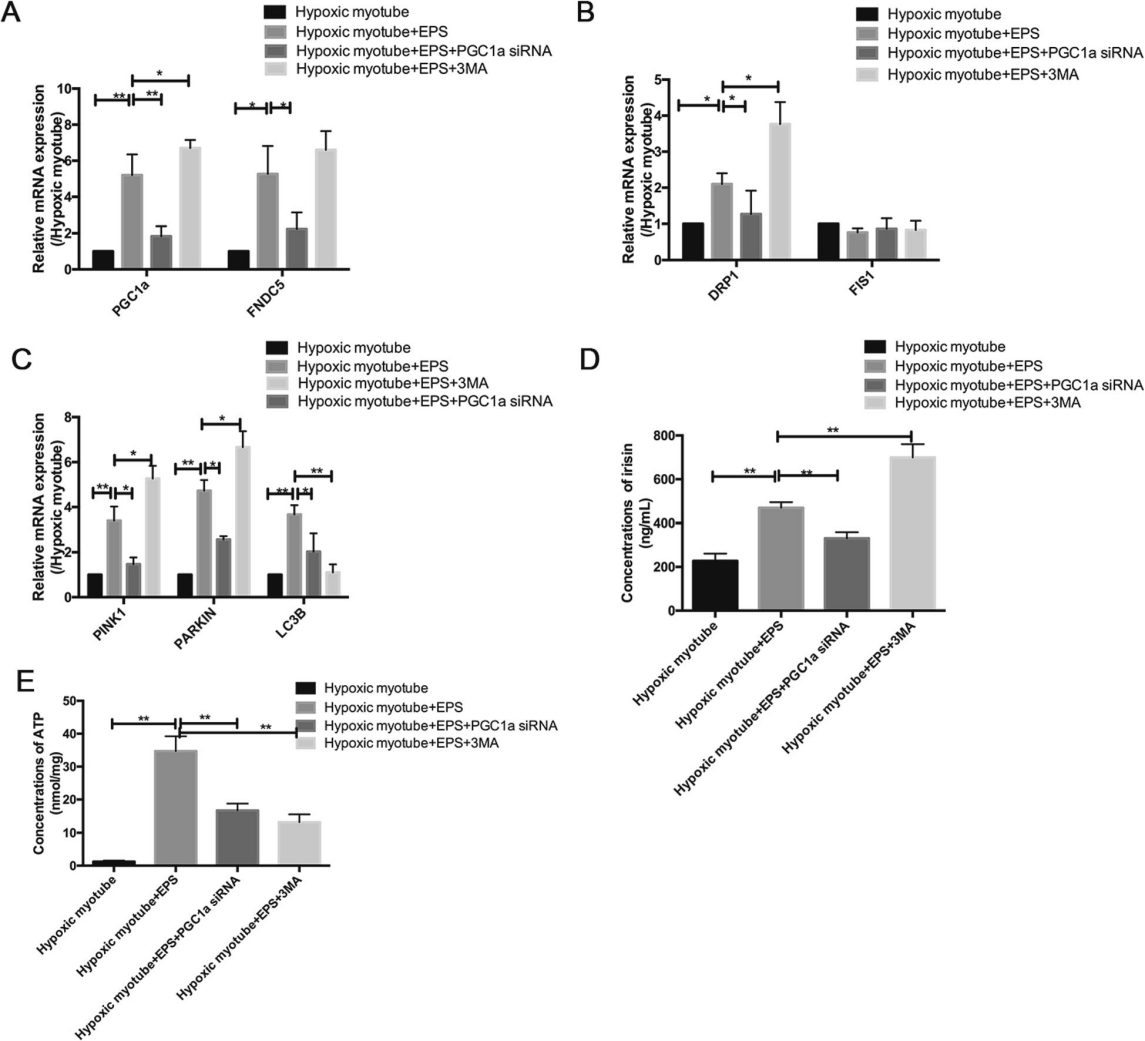
The relationship between PGC1a/FNDC5/irisin and mitochondrial turnover. **a** The comparisons of relative mRNA expression of PGC1a and FNDC5 among groups. **b** The comparisons of relative mRNA expression of mitochondrial dynamic markers among groups. **c** The comparisons of relative mRNA expression of mitophagy markers among groups. **d** The irisin concentrations in culture medium in 4 groups. **e** The comparisons of ATP concentrations among groups. *n* = 5 samples/group, *p < 0.05, **p<0.01

In vitro, PGC1a/FNDC5 mRNA expression in hypoxic and nutrient-deprived C2C12 myotube and the irisin levels in culture medium significantly increased in response to EPS stimulation (both *p* < 0.01). DRP1 and LC3B mRNA expression was also elevated following EPS (both *p* < 0.05) (Fig. 6a).

The downregulation of PGC1a by siRNA induced a decrease in FNDC5/irisin expression (both *p* < 0.05), restoring the effect of EPS on mitochondrial fission and mitophagy, as indicated by the downregulated expression of DRP1, PINK1, PARKIN, and LC3B (both *p* < 0.05), which results in the decreased total ATP production (Fig. 6a–e). The inhibition of autophagy by 3MA caused an increase in PGC1a expression and irisin concentrations (both *p* < 0.05), inducing the accumulation of upstream markers of mitophagy and mitochondrial fission markers, as indicated by the upregulated expression of PINK1, PARKIN, and DRP1, which suggests that exercise could regulate CLI via selective macro-autophagy (type 1 mitophagy) mediated by PINK1/PARKIN (Fig. 6a–d). Accordingly, the total ATP production was also impaired by the use of 3MA (Fig. 6e).

Interestingly, linear correlations of the irisin levels with the mRNA levels of PGC1a, FNDC5, PINK1, PARKIN, and DRP1 were also shown in the Pearson correlation analysis (all *p* < 0.05) (Supplementary Fig. 1).

## Discussion

Previously, exercise was seldom applied in the treatment of CLI due to concerns about aggravating limb ischemia [25]. A recent study has proposed that CLI patients can benefit from optimal exercise therapy following therapeutic angiogenesis [30]. Regarding the application of exercise in CLI, however, evidence is still limited. We have confirmed the efficacy of exercise in accelerating the recovery of skeletal muscle function and perfusion in elderly mice with CLI. For the first time, we revealed that exercise could increase mitochondrial fission and selective autophagy (mitophagy) to improve mitochondrial function and decreased cell apoptosis following CLI, which may be mediated by the PGC1a/FNDC5/irisin pathway.

In our study, the perfusion and skeletal muscle function of both the exercised and sedentary mice began to recover from day 14 after artery ligation. Although C57BL/6 mice are resistant to ischemia, chronic hypoperfusion symptoms lasting for 2 weeks confirmed the stability of our elderly murine model of CLI. Notably, exercise intervention significantly accelerated the recovery of function and perfusion, inducing decreased myopathic characteristics and elevated endothelial angiogenesis of the ischemic limbs in the exercised mice. Since moderate exercise is recommended for elderly patients with cardiovascular disease, exercise training with an intensity of 75% VO2 max was used in this experiment [16]. As none of the mice died during the training, we demonstrated the efficacy and safety of moderate exercise applied in CLI in elderly individuals.

Chronic hypoperfusion-induced myopathy converges on mitochondrial dysfunction. Lower ATP production and mitochondrial membrane potential were observed in the ischemic limbs than the controls in this study. As mitochondria are detrimental for the maintenance of the activity of skeletal muscle and regulation of cellular longevity [22], we revealed that exercise can significantly decrease the apoptosis of skeletal muscle cells possibly through improving mitochondrial function. In addition to the enhanced cellular longevity, electron transmission microscopy showed the more regular structure of sarcomeres in the exercised ischemic limbs, which ensures locomotion. Previous data have demonstrated that exercise protected skeletal muscle fibers from the long-term damage caused by ischemia/reperfusion [34]. We add to the literature and illustrate the potential protective role of exercise on mitochondrial function induced by CLI.

When mitochondria are exposed to stress, mitochondrial fission and mitophagy play dispensable roles in regaining metabolic homeostasis and maintaining mitochondrial integrity. As the expression of the mitochondrial fission marker DRP1 significantly decreased in the ischemic limbs, the difference in the expression of mitophagy markers (indicated by PINK1, PARKIN, LC3B) between the ischemic limbs and the controls did not reach significance in our study, indicating that mitochondrial damage induced by CLI may outweigh the ability of selective cleavage and clearance. The abnormal elongated intermyofibrillar mitochondria and the myeloid body formed by damaged mitochondria that accumulated in the fractured sarcomeres in sedentary ischemic limbs under electron transmission microscopy in our study further supports this view.

Interestingly, moderate exercise contributed to the elevation of DRP1 expression and the decreased mitochondrial length in the ischemic limbs in the current study, with no significant change in mitochondrial fusion marker (mfn2) expression, suggesting that the beneficial role of exercise in mitochondriopathy following CLI may be through the regulation of mitochondrial fission rather than fusion. The decreased FIS1 expression in the exercised ischemic limbs can be explained by the increased binding of DRP1 from the cytoplasm with FIS1 in the outer membrane of mitochondria, allowing the formation of autophagosomes and proper clearance [33].

In addition, more formation of mitochondrial derived vesicle (MDV)-like structures adjacent to mitochondrial membrane, the character of type3 mitophagy (microautophagy), was observed in exercised ischemic limbs, indicating that exercise may regulate CLI mainly through micromitophagy. Recent data has revealed that MDV can transport lipid and oxidative protein to lysosomes [31]. As mitochondria can serve as the membrane origin of the formation of autophagic vesicles, Cook et al. further provided the visual proof and showed that mitochondrial membranes contributed to the formation of membrane encapsulated autophagosome-like vesicular structure via measuring mitochondria staining positive for LC3 under electron microscopy, which indicates the efficient mitochondrial quality control process to recycle the dysfunctional mitochondria into autophagosomes [4, 9]. Although the MDV pathway still remains to be illustrated, the upregulated expressions of PARKIN accompanied by increased formation of vesicular structures in exercised ischemic limbs in this study could suggest the involvement of PARKIN in the micromitophagy, which was also consistent with the previous study [21]. Based on the above results, we can speculate that exercise may enhance micromitophagy in CLI.

As type 3 mitophagy is independent of DRP1-mediated mitochondrial fission, the upregulated DRP1 expression and mitochondrial fission in our study may indicate that exercise can promote the combination of type 1 and type 3 mitophagy. The rare formation of typical mitophagosomes and autolysosomes observed in ischemic limbs or normal controls can be explained by the fact that we assess the effect of exercise on mitochondrial quality control on day 30 after artery ligation when oxidative stress has become milder, considering that type 3 mitophagy is often induced by comparatively mild stress [17]. Besides, decreased mitochondrial response of elderly individuals to exercise stimuli could also account for the phenomenon [14]. Therefore, while the selective autophagy pathway underlies the regulative mechanism of exercise in CLI, microautophagy might be induced to a greater extent by exercise in elderly individuals during the late recovery period of CLI.

Despite exercise-increased mitophagy in the control limbs, the DRP1 and MFN2 levels were both downregulated, which can be attributed to aging-related suppression of the proteins involved in mitochondrial turnover [12]. While aging attenuated the response of mitochondria to exercise [14], CLI can exacerbate exercise-induced mitochondrial adaptations. Therefore, exercise can regain metabolic homeostasis by stimulating beneficial mitochondrial adaptation in CLI in elderly individuals.

Consistent with the exercise enhanced mitochondrial fission and selective autophagy in ischemic limbs, the more induced PGC-1a in exercised ischemic limbs further supports the involvement of PGC1a in the influence of exercise on mitochondrial adaptations in CLI. Although the previous study has reported that PGC1a may improve CLI by regulating antioxidant defense and mitohormesis [16], limited studies have focused on the mechanistic role of PGC1a in mitochondrial turnover, considering the importance of mitochondrial fission and mitophagy in regaining mitochondrial homeostasis under pathological conditions such as CLI. FNDC5/irisin is mainly expressed and secreted by skeletal muscle, which is activated by the upstream marker PGC1a [37]. In addition to the regulation of glucose and lipid metabolism, FNDC5/irisin could protect mitochondrial function in cardiomyocytes against ischemia/reperfusion injury [35]. As we used EPS to mimic exercise stimuli in vitro, the downregulated expression of PGC1a/FNDC5/irisin can aggravate the EPS-increased mitochondrial fission and mitophagy and impede the total ATP production in ischemic limbs, which expands the role of PGC1a/FNDC5/irisin in exercise-induced mitochondrial turnover. The EPS-induced accumulation of PGC1a/FNDC5/irisin and mitophagy markers (indicated by PINK1, PARKIN) after inhibiting autophagy further supports that exercise may regulate CLI through selective autophagy via PGC1a/FNDC5/irisin pathway. Based on the positive association between the irisin levels in the culture medium and the markers of mitochondrial turnover in this study, we can speculate that the mitochondrial dynamics in hypoxic and nutrients deprived myotubes may be regulated by the paracrine or autocrine action of FNDC5/irisin initiated by exercise-induced PGC1a.

There are several limitations to our study. First, we have not assessed the dynamic process of mitochondrial turnover in vivo. The regulative mechanism of exercise on micromitophagy in elderly individuals with CLI still remains to be illustrated. Second, although the decreased total ATP production accompanied by the downregulated mitochondrial membrane potential could partly reflect the effect of aerobic exercise on mitochondrial function in this study, a more proper method like the assessment of oxygen consumption rate cannot be conducted in the current study. Third, although sequential ligations were previously used to induce a sustainable chronic CLI model, such a method cannot be successfully achieved in elderly mice because of the comparatively high risk of the operation, infection, and anesthesia. However, the stability of the CLI model in elderly animals in our study was confirmed based on the assessment of perfusion and function.

However, we have several strengths. First, as elderly populations are more susceptible to CLI, we added to the literature by revealing the effect and safety of exercise in elderly CLI mice, which may provide a potential treatment method for elderly patients with CLI who are often ineligible for surgical and endovascular revascularization and decline to join exercise rehabilitation programs due to safety concerns. Second, for the first time, we conducted in vivo and in vitro studies to reveal that exercise-induced mitochondrial turnover in CLI could be through the paracrine or autocrine action of FNDC5/irisin activated by PGC1a.

## Conclusions

In conclusion, exercise can effectively enhance the recovery of function and perfusion and decrease apoptosis in elderly individuals with CLI. Moreover, the exercise-increased mitochondrial fission and selective autophagy (mitophagy) in ischemic limbs can be initiated by the PGC1a/FNDC5/irisin pathway. Our findings confirmed the efficacy of exercise therapy in CLI in elderly individuals and indicate the potential of targeting PGC1a/FNDC5/irisin as the exercise-replacement method for patients with limited exercise capacity, which provides new strategies for the treatment of CLI.

## Supplementary information

**Additional file 1: Supplementary Fig. 1.** The associations between irisin concentrations in culture medium and the mitochondrial turnover markers.

## Data Availability

The data that support the findings of the study are available from the corresponding author upon reasonable request.
